# Differentiating Childhood Traumas in Inflammatory Bowel Disease

**DOI:** 10.1093/jcag/gwad026

**Published:** 2023-08-28

**Authors:** Lauren Gnat, Valentina Mihajlovic, Krista Jones, Dean A Tripp

**Affiliations:** Department of Psychology, Queen’s University, Kingston, Ontario, Canada; Department of Psychology, Queen’s University, Kingston, Ontario, Canada; Department of Psychology, Queen’s University, Kingston, Ontario, Canada; Departments of Psychology, Anesthesiology and Urology Queen’s University, Kingston, Ontario, Canada

**Keywords:** inflammatory bowel disease (IBD), healthy controls (HC), childhood trauma, confiding, resilience, depressive symptoms

## Abstract

**Background:**

Inflammatory bowel disease is characterized by chronic inflammation of the gastrointestinal tract. Research on inflammatory bowel disease has shown a connection to childhood traumatic events. However, few studies have focused on specific types of traumatic experiences and the impact of confiding in others on disease-related outcomes. This comparative, cross-sectional study expected that: (1) patients would report higher prevalence rates of childhood traumas than healthy controls; (2) healthy controls would report fewer and less severe traumatic experiences than patients and less confiding in others compared to patients; (3) childhood trauma severity would be indirectly related to depressive symptoms through resilience and confiding in others would moderate this relationship.

**Methods:**

Participants completed an online survey; an inflammatory bowel disease patient group (*N* = 195, *M*_age_ = 40.48, 76.4% female) was compared to a similarly recruited sample of healthy controls (*N* = 190, *M*_age_ = 31.16, 59.5% female).

**Results:**

Patients reported a higher prevalence of experiencing sexual traumas (*P* = .031), major upheavals (i.e., disruptions) (*P =* .048), and violence (*P* = .050) than controls. Patients had significantly higher total trauma severity odds ratios (OR 0.89, 95% CI[0.81,0.97]) and significantly lower total confiding in other odds ratios than controls (OR 1.09, 95% CI[1.02,1.16]). Childhood trauma severity was indirectly related to depressive symptoms through resilience, *b =* .05, SE *=* 0.09, 95% CI[0.01,0.09]; however, confiding did not moderate this relationship.

**Conclusions:**

Patients reported more sexual, disruptive, and violent traumas. Although confiding did not act as a moderator, trauma was related to depressive symptoms through resilience.

## Introduction

Inflammatory bowel disease (IBD) is a widespread chronic disease that affects more than six million individuals worldwide.^1^ IBD encompasses the subtypes of Crohn’s disease (CD) and ulcerative colitis (UC) and is characterized by chronic inflammation of the gastrointestinal tract, exhibiting a relapsing and remitting course. Symptoms include abdominal pain, nausea, diarrhoea, bacterial contamination, swelling, joint and skin problems, and fatigue.^1–3^ The brain–gut axis model may explain the disease development of IBD and comorbid psychopathology^3^; individuals who endure childhood traumas may experience adverse gut microbiome changes leading to exaggerated and dysfunctional immune responses and inflammation.^3–5^ Childhood traumatic experiences have been linked to inflammation and immune dysfunction, suggesting vulnerability to developing mental and somatic disorders in adulthood.^6–8^ Specific childhood traumas (i.e., physical or sexual abuse) have been linked to depression in IBD.^4,9,10^ However, the relationship between trauma and IBD is not strictly causal, as other factors like genetics, smoking, stress, and malnutrition could also contribute.^2,5,7^

Few studies isolate specific traumas in IBD and fewer contrast that data with controls.^4,6,8^ Compared to healthy controls, patients with IBD report a higher prevalence of overall stress, with elevated interpersonal violence experiences.^4,6^ Recent abdominal pelvic research outside of IBD examined childhood traumas between patients with irritable bowel syndrome (IBS) and healthy controls. The total traumatic experiences and total trauma severity were associated with increased odds of IBS, whereas confiding (i.e., actively disclosing a traumatic event to another person) was associated with decreased odds of IBS.^11^ Although IBS and IBD diagnoses share similar symptoms, IBD comes with additional concerns, such as surgeries and physical complications that would be rare for patients with IBS.^6,11^ No research examines whether trauma count, severity, or confiding could influence IBD odds. Thus, an extension study of unexamined types of traumas experienced by individuals with IBD was proposed to augment this previous research and include protective factors such as confiding in others about traumatic events.

Research has shown that childhood traumas have been linked to depression in individuals with IBD; the association with depression is more robust in those with IBD who experienced childhood trauma than in the general IBD population.^4,12^ Depression can worsen the disease course in IBD by exacerbating IBD symptoms, reducing the patient’s quality of life, and decreasing pain tolerance.^9,12^ While there is no causal evidence linking childhood trauma to IBD, trauma could be more prevalent in IBD populations due to its connection to inflammation, which increases the risks of inflammatory diseases like IBD.^4^ Within the IBD population, those who reported enduring childhood trauma experienced more severe depressive symptoms than those who did not report trauma.^8,9,12^ Patients who endured childhood trauma often report lower levels of resilience (i.e., the ability to recover from and overcome stressful events) than the general IBD population.^1,8^ Resilience can mediate the relationship between childhood trauma and depression, where more severe childhood trauma is associated with lower resilience, which is related to more severe depressive symptoms.^1,8,10^ Confiding about one’s trauma may influence the relationship between childhood trauma and depression. Previous research suggests that confiding in others after trauma can be linked to better mental health and reduced gastrointestinal symptomology for those with IBS.^11,13^ Opening up about trauma and expressing emotions may reduce psychophysiological tension, potentially leading to fewer or less severe depressive symptoms.^1,14^ It remains unknown whether confiding could enhance resilience and protect against depressive symptoms in IBD patients who experienced childhood trauma.

The current cross-sectional study had three aims and corresponding hypotheses. The first aim was to assess the prevalence of six types of childhood traumas, as measured by the Childhood Traumatic Events Scale (CTES), across IBD and healthy controls.^13^ These six types of traumas include the death of a loved one, parental upheaval, sexual trauma, violence trauma, severe illness or injury, and other major upheavals.^13^ We predicted that patients with IBD would report more traumas than controls. The second aim was to examine the frequency, severity, and amount of confiding associated with the six traumas. We expected that the frequency and severity of the childhood traumas would predict higher odds of belonging to the IBD group, whereas confiding to others would predict higher odds of a healthy control group membership. Lastly, the third aim was to determine if confiding acts as a buffer between childhood trauma and depression when mediated by resiliency in the IBD sample. We hypothesized that a positive association between childhood trauma and depressive symptoms^10^ would occur indirectly through resiliency, with confiding moderating this relationship.

## Methods

### Participants

In five months in 2019, 195 participants in the IBD sample (median age 40.00, IQR 40.0, SD 12.67) were recruited for an online survey through communication platforms (e.g., Facebook groups). Eligible participants were fluent in English, above 18 years old, and self-reported having an IBD diagnosis. Exclusion criteria included significant medical conditions (e.g., severe pulmonary, cardiac, or renal disease), any GI diseases (e.g., celiac and IBS) and severe psychiatric illness (e.g., psychosis).

Over two months in 2021, 190 healthy controls (median age 27.00, IQR 27, SD 11.52) were recruited through online communication platforms (e.g., Facebook groups and Instagram). Participants were fluent in English and above 18 years old. Exclusion criteria included a significant medical condition or psychiatric disorder, current or history of chronic pain syndrome or disorder, previous IBD diagnosis, presence of IBD symptoms, and notable GI or other diseases, disabilities, or mobility impairments.

### Measures

All participants provided their age, gender, ethnicity, country of residence, education level, and employment status to match the samples based on similar characteristics.

Patients’ IBD-related symptoms (e.g., bowel issues, fatigue, and abdominal discomfort), date of diagnosis, surgeries, and treatments received were assessed using the 26-item measure IBD Symptom Inventory Short Form (IBDSI-SF; α = 0.94).^15^ Healthy controls completed the IBDSI-SF questionnaire to confirm the absence of IBD-related symptoms. Total scores ranged from 0 to 95, with higher scores indicating high symptom severity.

Childhood trauma prevalence, count, severity, and confiding for six traumas were measured using the Childhood Traumatic Events Scale (CTES; α = 0.83). The CTES assessed participants’ self-reported traumatic experiences before age 17 and the extent of confiding.^13^ Trauma count scores range from 0 to 6, with higher scores indicating experiencing more traumas. Trauma severity and confiding were each comprised of six items, ranging from 6 to 42, with higher scores indicating higher trauma severity or amount of confiding, respectively.

Depressive symptoms were screened using the Patient Health Questionnaire (PHQ-9; α = 0.87), a nine-item self-report measure based on the Diagnostic and Statistical Manual of Mental Disorders Fourth Edition criteria for depression.^16^ Participants rated items on a four-point scale ranging from 0 (*not at all*) to 3 (*nearly every day*). Total scores ranged from 0 to 27, with higher scores indicating more severe depressive symptoms.

The Brief Resilience Scale (BRS; α = 0.84) includes six items capturing one’s ability to bounce back from stressful life events.^17^ Participants rated items on a five-point scale ranging from 1 (*strongly disagree*) to 5 (*strongly agree*). A total score was calculated by averaging the six items; totals ranged from 1 to 6, with higher scores indicating higher levels of resilience.

### Procedure

Ethics clearance was provided by the Queen’s University Health Sciences Research Board for both data collection periods. The IBD patient data were drawn from a more extensive study investigating suicide risk predictors in IBD.^18^ Individuals with IBD were recruited through support groups and advertisements on Facebook and the Crohn’s and Colitis Canada website. The control data was collected for this study, with controls recruited through social media platforms (e.g., Facebook groups and broadly and Instagram). Online recruitment allowed us to reach a broader participant pool beyond the local area of Kingston, Ontario, which has a predominantly White population—additionally, recruitment for the controls needed to mirror the recruitment approach used for the comparison patient group. The links to access the surveys were included in online postings. Surveys were administered anonymously using Qualtrics, and participants provided consent after reading the letter of information. Skipping survey items had no penalty. Debriefing materials with resources for distress were provided after study completion. The questionnaires took approximately 10 min to complete.

### Statistical analysis

Data from the samples were combined into a single data set. The data was reviewed for missing or impossible values, and responses missing over 15 percent of the data or containing impossible values were deleted. Total and subscale scores were calculated based on measurement specifications.

Statistical analyses were executed using version 27 of SPSS software.^19^ Chi-square tests and *t*-tests assessed differences across demographic variables and trauma prevalence counts for the six traumas measured in the CTES. Logistic regression determined the odds ratios for trauma count, trauma severity, and confiding in predicting group membership (i.e., IBD or healthy controls). Model 4 of Hayes’^19^ PROCESS macro for SPSS tested the mediation of whether resilience mediated the association between childhood trauma and depressive symptoms. The moderated mediation, where confiding moderated the mediation model, was tested using Model 8 of Hayes’^19^ PROCESS macro for SPSS.

## Results

Out of the initial 258 healthy control participants, 68 were excluded for completing less than 85percent of the survey. In the patient sample, 85 participants were excluded for incomplete responses, and 143 were excluded for not residing in North America.

### Demographic analyses

Table 1 shows significant differences between patients (*N* = 195) and healthy controls (*N* = 190) for age, gender identity, racial-ethnic identity, country of residence, and employment status. Patients were older than healthy controls, *t*(1,383) = 7.53, *P* < .001. There were more women in the IBD group compared to healthy controls and more men and non-binary individuals in the healthy control group compared to the IBD group, χ^2^ = (2, *N* = 385) = 16.85, *P* < .001. Across racial–ethnic identity, controls had fewer White participants and more Hispanic, Latino, and Asian participants than patients, χ^2^ = (5, *N* = 385) = 24.56, *P* < .001. The control group had fewer participants from Canada and more from the United States than the patients, χ^2^ = (2, *N* = 385) = 12.00, *P =*.002. More patients described their employment status as unemployed or disabled compared to the healthy controls, and there were more students among controls than patients, χ^2^ = (4, *N* = 385) = 42.29, *P* < .001.

**Table 1.** Differences in demographic characteristics across healthy controls and IBD patients.

Sociodemographic group	HC *N* = 190 *N* (%)	IBD *N =* 195 *N* (%)
Age (*M*, SD)	31.16, 11.52	40.48, 12.67^***^
Gender identity		
Male	70 (36.8)	46 (23.6)^**^
Female	113 (59.5)	149 (76.4)^***^
Other, please specify…	7 (3.7) ^**^	
Race/ethnicity		
Caucasian/White	151 (79.5)	183 (93.8)^***^
Hispanic or Latino Aboriginal	11 (5.8) 1 (0.5)	1 (0.5)^**^ 1 (0.5)
Asian	17 (8.9)	2 (1.0)^***^
Black/African Canadian	5 (2.6)	2 (1.0)
Other	5 (2.6)	6 (3.1)
Country of residence		
Canada	107 (56.3)	141 (72.3)^***^
United States Other	81 (42.6) 2 (1.1)	54 (27.7)^**^
Education		
Less than high school	2 (1.1)	3 (1.5)
High school or GED	17 (8.9)	18 (9.2)
Some college/university	57 (30.0)	46 (23.6)
College/university graduate	59 (31.1)	86 (44.1)
Some graduate or professional school after college/university	22 (11.6)	11 (5.6)
Graduate from graduate or professional school after college/university	33 (17.4)	31 (15.9)
Employment status		
Employed	125 (65.7)	124 (63.6)
Unemployed	12 (6.3)	26 (13.3)^*^
Retired	5 (2.6)	12 (6.2)
Disabled		17 (8.7)^***^
Student	48 (25.3)	15 (7.7)^***^
Missing		1 (0.5)
Type of IBD diagnosis received		
Crohn’s Disease Ulcerative Colitis		112 (57.4) 74 (37.9)
Both		9 (4.6)

*Note:* HC = healthy controls; IBD = inflammatory bowel disease. *p < .05. **p < .01. ***p < .001.

### Objective 1: trauma prevalence across healthy controls and IBD patients

**Figure 1.**
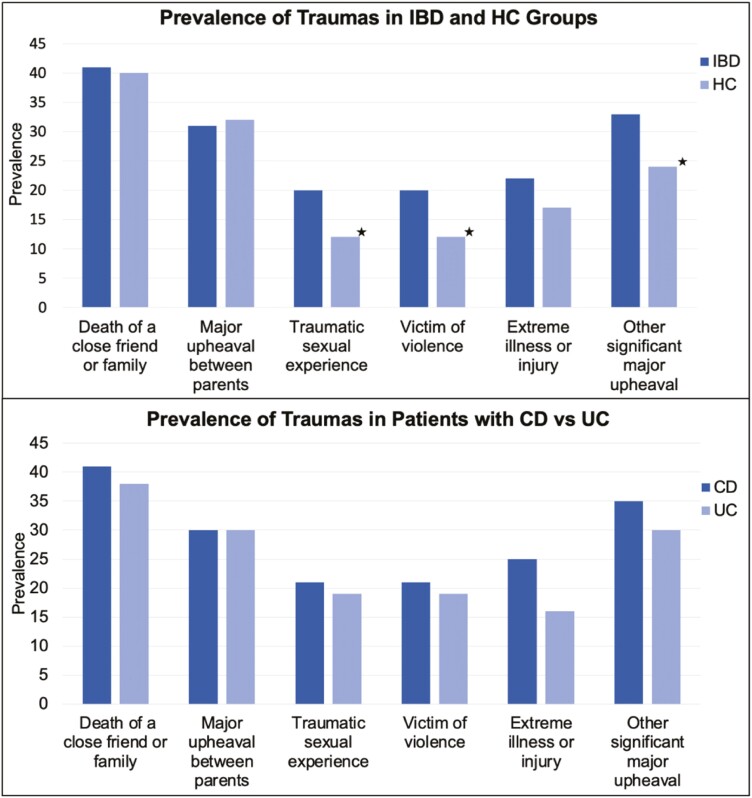
Trauma prevalence across healthy controls and IBD. *Note:* The frequency count for childhood traumatic events across the HC and IBD groups.

As shown in figure 1, there were significant differences in the prevalence of childhood traumas across healthy controls and patients who experienced sexual traumas, χ^2^ = (1, 384) = 4.67, *P =*.031, being a victim of violence, χ^2^ = (1, 385) = 3.81, *P =*.050, and other major upheavals, χ^2^ = (1, 385) = 3.90, *P =*.048; in general, patients with IBD experienced sexual, violent, and disruptive traumas more than controls.

### Objective 2: trauma variables associated with IBD status

A logistic regression model was used to determine the odds ratios for IBD status, depending on trauma count, trauma severity, and confiding about one’s trauma (see Table 2). The IBD group was the reference group; the column depicts the likelihood of being in the IBD group, depending on the trauma variables.

**Table 2.** Odds ratios for IBD group membership.

HC vs. IBD	OR (95% CI)
Total scores	
Total number of traumas	1.28 (.74–2.19)
Total trauma severity	0.89 (.81–0.97)^*^
Total confiding scores	1.09 (1.02–1.16)^**^
**Trauma event**	
Death of a close friend or family	0.95 (0.62–1.43)
Major upheaval between parents	1.35 (0.84–2.17)
Traumatic sexual experience	0.62 (0.34–1.14)
Victim of violence	0.69 (0.37–1.28)
Extreme illness or injury	0.82 (0.49–1.38)
Other significant major upheaval	0.70 (0.43–1.14)
**Trauma severity**	
Death of a close friend or family	0.99 (0.90–1.09)
Major upheaval between parents	1.04 (0.94–1.15)
Traumatic sexual experience	0.94 (0.84–1.05)
Victim of violence	0.90 (0.80–1.03)
Extreme illness or injury	0.91 (0.81–1.02)
Other significant major upheaval	0.96 (0.87–1.05)
**Confiding**	
Death of a close friend or family	1.02 (0.92–1.14)
Major upheaval between parents	1.11 (0.97–1.27)
Traumatic sexual experience	0.88 (0.71–1.08)
Victim of violence	0.98 (0.82–1.18)
Extreme illness or injury	0.96 (0.85–1.10)
Other significant major upheaval	0.99 (0.86–1.13)

*Note:* HC = healthy controls; IBD = inflammatory bowel disease.*p < .05. **p < .01. ***p < .001.

Overall, the model fit for the total scores was significant, *X*^2^(3) = 16.373, *P* < .001, Nagelkerke’s *R*^2^ = 0.056, Cox & Snell *R*^2^ = 0.042. Total trauma count, *b* = 0.124, SE = 0.244, *P* = .611, was not significantly associated with IBD status. However, total trauma severity *b* = –0.106, SE = 0.045, *P* = .017, and total trauma confiding, *b* = 0.086, SE = 0.033, *P* = .008, were associated with group membership. Trauma count, severity, and confiding scores for each of the six types of traumas were not significantly associated with IBD status (*P* > .05).

### Objective 3: moderated mediation analyses for IBD

**Figure 2.**
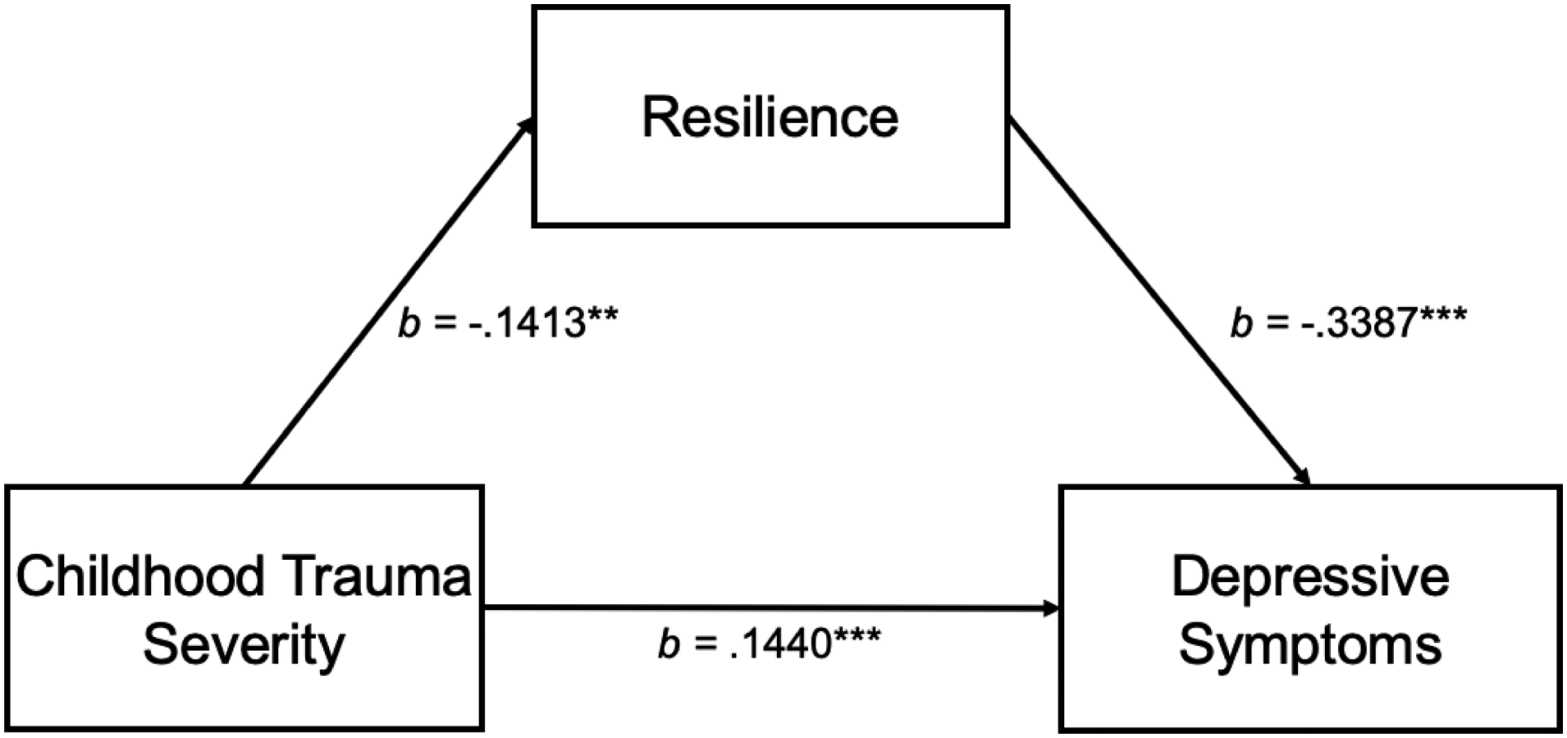
Resilience as a mediator of childhood trauma severity and depressive symptoms. *Note:* Mediation model is tested by Model 4 of the PROCESS macro. ^*^P < .05. ^**^P < .01. ^***^P < .001.

Model 4 used 95 percent confidence intervals (CIs) and 10,000 bootstrap samples. As shown in figure 2, the indirect, direct, and total effects of childhood trauma severity on depressive symptoms through resilience were significant: indirect effect, *b* = 0.05, SE = 0.09, 95% CI[0.01,0.09], direct effect, *b* = 0.14, SE = 0.04, 95% CI[0.07,0.22], and total effect, *b* = 0.19, SE = 0.04, 95% CI[0.12,0.27].

Model 8 also used 95% CIs and 10,000 bootstrap samples. The direct effect of resilience on depressive symptoms was significant, *b* = –0.34, SE = 0.05, 95% CI[–0.43, –0.24]. The direct effect of trauma severity on depressive symptoms was significant, *b* = 0.14, SE = 0.04, 95% CI[0.05,0.22]. Confiding was not a significant moderator of the relationship between trauma severity and resilience, *b* = 0.003, SE =0.01, 95% CI[–0.01,0.01] or trauma severity and depressive symptoms, *b* = 0.01, SE = 0.003, 95% CI[–0.002,0.01]. The overall model was not supported by the index of moderated mediation, –0.001, SE = 0.003, 95% CI[–0.005,0.007].

## Discussion

This study explored the impact of specific traumas on patients with IBD compared to healthy controls and examined the role of confiding in buffering against depressive symptoms. Consistent with previous research,^6^ patients with IBD reported more childhood sexual, disruptive, or violent traumas than healthy controls. Previous research suggests that experiences of childhood sexual abuse, physical abuse, and parental upheavals can result in a higher frequency of medical visits for symptoms associated with IBD.^20^ Healthcare providers who understand the effects of trauma on the patient’s emotional and physical health can customize their care to exhibit empathy, sensitivity, foster a safe environment and open communication, and provide treatment that puts the patient at the centre.^21^ These trauma-informed practises can improve patient-provider relationships, patient engagement, treatment adherence, and health outcomes while reducing avoidable care and costs for healthcare and social service sectors.^21^ Thus, these findings highlight the importance of clinicians asking about childhood traumatic experiences in IBD care.^20,21^

### Trauma variables associated with IBD status

Patients reported more severe childhood trauma than controls. This finding may partially be due to the psychosomatic influence of early traumatic experiences.^5^ Traumatic events in childhood can lead to heightened stress responses, weakening the inflammatory response in the gastrointestinal tract and leaving individuals potentially vulnerable to gastrointestinal dysfunction.^3,5,11^ Further, patients with IBD confided in others about their traumatic experiences less than controls. Confiding can be a cathartic process of expressing emotion. Releasing the negative emotion through disclosing the trauma to a trusted confidant can reduce the physiological and psychological stress of inhibiting negative emotions and promote positive outcomes, such as connecting with others to gain social support.^13,22^ Research examining why patients reported sharing their traumatic experiences less than healthy controls is warranted.

### Moderated mediation analyses for IBD

Although our proposed model does not support confiding buffering against depressive symptoms, this study highlights the association between childhood trauma severity and depressive symptoms and the mediating role of resilience.^10^ Contextual influences in childhood, such as self-regulation, social support, and positive attachments, contribute to the development of resilience.^1,10,12^ Severe traumas can hinder childhood resilience development, leading to lower social support, adaptive coping, and self-efficacy, contributing to more depressive symptoms from childhood to adulthood.^1,10,23^ Fostering high resilience in patients with IBD could decrease the risks of depression by teaching productive coping strategies, readjusting negative self-beliefs, and connecting individuals to social support when managing IBD symptoms.^1,10,23^ Contrary to expectations, confiding did not moderate the relationship between trauma severity and resilience, nor between trauma severity to depressive symptoms. Past literature suggests that social support could reduce patients’ psychological distress associated with IBD.^1,9^ Confiding can be conceptualized as seeking social support by expressing negative emotions.^13,22^ Given that the current study did not measure crucial factors associated with confiding (e.g., depth, confidant’s response, and emotional response), confiding experiences could have been negative for patients.^24^ As in previous research, patients who received undesirable reactions from their social contacts (e.g., pity, overreacting, and sadistic empathy [i.e., supporter derives pleasure from the patient’s pain]) felt worse after receiving social support.^24^

### Clinical implications

This study offers vital information about the most frequent and harmful childhood traumas experienced by patients with IBD. Addressing these traumas through psychotherapeutic interventions may help patients manage their conditions since heightened stress from traumatic events is associated with worsened disease symptomology and flare-ups.^7,20,23^ The current findings suggest exploring confiding further in patients with IBD, considering that healthy controls confided significantly more than IBD patients. This difference may represent a potential clinical opportunity because confiding could have a cathartic influence in releasing trauma-related physiological tension.^11,13^ Confiding less in others could put individuals at risk for behaviours related to developing IBD, such as maladaptive coping strategies (e.g., rumination and emotional suppression) and low social support seeking.^10,11,13,23^ Confiding did not protect against developing depressive symptoms in IBD, so clinical awareness regarding interpersonal openness might be important for treating depression in this population.

### Limitations and future directions

The healthy controls and patients in the samples differed significantly in age, gender, race, and country of residence. Moreover, most participants were White Canadian women. Despite these demographic differences, the control sample includes more diversity, and having more diverse samples is vital in increasing the study’s generalizability. Further, the cross-sectional analysis does not allow for causal inferences. Longitudinal designs are needed to establish a causal relationship between IBD symptomology, depressive symptoms, and childhood trauma over time.^25,26^ Finally, online recruitment may introduce self-selection sampling bias, as individuals seeking health information online may differ from the general population of chronic disease patients.^27,28^ The study had strengths, such as using reliable and valid measures to ensure consistency with previous studies. Furthermore, the substantial sample size increased confidence in the findings by reducing the margin of error and enhancing statistical power.

## Conclusion

This research offers valuable insights into the effects of specific traumatic events, including sexual, disruptive, and violent traumas, on patients with IBD compared to controls. The results suggest that low resilience may mediate the impact of childhood trauma severity on depressive symptoms. These findings highlight the importance of further examining the psychological impacts of traumatic events in individuals with IBD.

## Supplementary Material

gwad026_suppl_Supplementary_Material

## Data Availability

The article’s data will be shared on reasonable request to the corresponding author.

## Abbreviations:

BRS: Brief Resilience Scale
CD: Crohn’s Disease
CTES: Childhood Traumatic Events Scale
HC: Healthy Control
IBD: Inflammatory Bowel Disease
IBDSI-SF: IBD Symptom Inventory Short Form
IBS: irritable bowel syndrome
PHQ-9: Patient Health Questionnaire
SPSS: Statistical Package for the Social Sciences
UC: Ulcerative Colitis
